# The Soil–Plant Continuity of Rare Earth Elements: Insights into an Enigmatic Class of Xenobiotics and Their Interactions with Plant Structures and Processes

**DOI:** 10.3390/jox15020046

**Published:** 2025-03-20

**Authors:** Angela Martina, Lorenzo Ferroni, Elena Marrocchino

**Affiliations:** Department of Environmental and Prevention Sciences, University of Ferrara, 44121 Ferrara, Italy; mrtngl1@unife.it (A.M.); mrrlne@unife.it (E.M.)

**Keywords:** plant, soil, rare earth elements, hormesis, geochemistry, metabolism, toxicity, lanthanides, europium

## Abstract

Rare earth elements (REEs) are increasingly present in the environment owing to their extensive use in modern industries, yet their interactions with plants remain poorly understood. This review explores the soil–plant continuum of REEs, focusing on their geochemical behavior in soil, the mechanisms of plant uptake, and fractionation processes. While REEs are not essential for plant metabolism, they interact with plant structures and interfere with the normal functioning of biological macromolecules. Accordingly, the influence of REEs on the fundamental physiological functions of plants is reviewed, including calcium-mediated signalling and plant morphogenesis. Special attention is paid to the interaction of REEs with photosynthetic machinery and, particularly, the thylakoid membrane. By examining both the beneficial effects at low concentrations and toxicity at higher levels, this review provides some mechanistic insights into the hormetic action of REEs. It is recommended that future research should address knowledge gaps related to the bioavailability of REEs to plants, as well as the short- and long-range transport mechanisms responsible for REE fractionation. A better understanding of REE–plant interactions will be critical in regard to assessing their ecological impact and the potential risks in terms of agricultural and natural ecosystems, to ensure that the benefits of using REEs are not at the expense of environmental integrity or human health.

## 1. An Introduction to Rare Earth Elements

**Figure 1:**
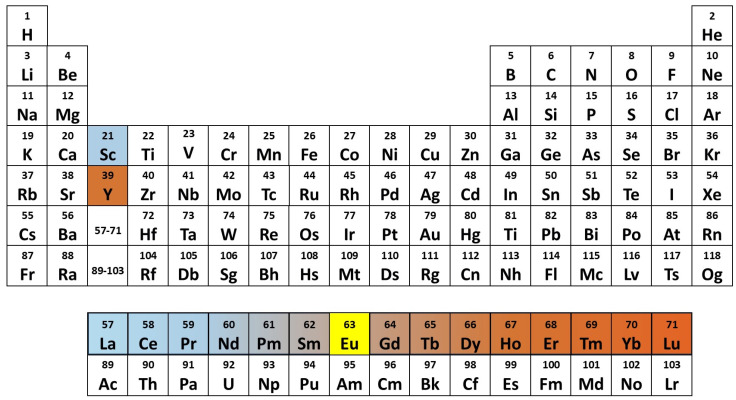
Periodic table highlighting the rare earth elements (REEs). The lanthanide series is depicted with a gradient of colors reflecting the lanthanide contraction, from La (a lower atomic number), assigned a blue color, to Lu (a higher atomic number), assigned a rust color. The elements Sc and Y are highlighted in colors based on their chemical affinity to the lanthanides, while Eu is colored yellow to emphasize its anomalous behavior.

Rare earth elements (REEs) encompass a group of 17 chemically similar elements, including 15 lanthanides, with atomic numbers ranging from 57 (lanthanum, La) to 71 (lutetium, Lu), as well as the lighter elements, scandium (Sc) and yttrium (Y), which exhibit comparable properties (Figure 1). While REEs are extensively utilized across various industries, their environmental behavior and biological effects are still not fully understood. Traditionally, research on REEs was primarily centered on their geochemical properties, extraction techniques, and industrial applications. However, their growing environmental presence, resulting from mining, industrial processes, and agricultural use, has raised concerns regarding their ecological impact and potential accumulation in food chains. Although previous studies have investigated the geochemical behavior of REEs in soils, their mobility, and their interactions with microorganisms, research on their uptake and distribution in plants remains incomplete. While some studies have investigated REEs in plant cells and organs and have generally interpreted their action as xenobiotic analogues of calcium, their physiological effects, ranging from beneficial to potentially toxic, are not yet fully understood. Furthermore, the mechanisms regulating their transportation, accumulation, and fractionation across different plant compartments continue to be a subject of debate.

This review presents a comprehensive synthesis of the existing knowledge on the soil–plant continuum of REEs, focusing on their mobility in the rhizosphere, mechanisms of uptake, short- and long-range transportation, and fractionation within plant organs. It explores their molecular interactions with plant cell structures and the influence of these interactions on physiological processes, such as calcium signalling, photosynthesis, and morphogenesis. Unlike previous literature review articles on REEs, this paper integrates insights from soil geochemistry with plant morphology, biochemistry, and physiology, without neglecting some relevant environmental cues, in order to provide an original multidisciplinary perspective on REEs as an emerging class of xenobiotics. By identifying gaps in the current understanding, it underscores the ecological implications of REEs and emphasizes the need for further research on their long-term impact on plant life and environmental integrity.

The major comprehensive reviews on REEs were used to guide the subsequent thorough search of the Elsevier Scopus and Google Scholar databases for publications dealing with the origin and status of REEs in soil and plants, their molecular interactions with plant cell structures, and their effects on plant processes. It became clear that the molecular interactions involving REEs that lead to beneficial or toxic effects largely overlap and, therefore, we found it more useful to focus on plant processes. Accordingly, after introducing REEs, this review is structured according to the following sections: Basic chemical information about REEs; Geochemistry of REEs in soils; Absorption and fractionation of REEs in plants; Impacts of REEs on plant processes; Conclusions and future directions for research.

The classification of these elements as “rare” stems from their sparse occurrence in concentrated, economically viable deposits, despite their widespread, albeit diffuse, presence in the Earth’s crust [1,2]. This unique combination of availability and dispersion makes them challenging to extract and refine, resulting in their designation as “rare earths” [3,4,5]. However, in absolute terms, REE concentrations can be comparable to or exceed those of more familiar metals, like copper (Cu) and zinc (Zn) [6,7]. Cerium (Ce) is the most abundant REE, ranking as the 25th most common element in the Earth’s crust.

The historical trajectory of REE discovery and applications reflects both the scientific and geopolitical significance of REEs, as they have been transformed from obscure elements to critical resources for modern technology and industry. The discovery and exploration of REEs has an extensive and complex history, dating back to the late 18th century.

The journey began in 1787, when Swedish chemist Carl Axel Arrhenius encountered a dense, black mineral near the village of Ytterby, Sweden, which would later be instrumental in the discovery of several REEs. Finnish chemist, Johan Gadolin, first analyzed this mineral in 1794, identifying a new “earth”, which he named “yttria”, after the site of its discovery. This mineral would become the foundation for isolating other REEs, because it contained a mixture of previously unknown elements [7]. The subsequent years saw the gradual isolation of elements from this and other rare earth sources, with Ce being identified in 1803 by Wilhelm Hisinger and Jöns Jakob Berzelius. Throughout the 19th century, chemists like Carl Gustaf Mosander and others continued to separate and identify additional REEs from complex mineral assemblages. By the early 20th century, most REEs had been discovered, with the final naturally occurring, heaviest, and rarest REE, Lu, identified in 1907. The set of known REEs was completed in 1945 with the discovery of promethium (Pm), an artificial radioactive element generated during nuclear reactions [8].

After their discovery, REEs have progressively gained significant scientific and industrial attention owing to their versatile applications across numerous sectors, including advanced technologies, green energy, agriculture, zootechny, and biomedicine [9]. They are indispensable in manufacturing high-strength permanent magnets, phosphors for displays, rechargeable batteries, catalytic converters, and various components for renewable energy technologies, such as wind turbines and electric vehicles. Furthermore, REEs are essential in defense applications, where their unique magnetic and conductive properties play a role in precision-guided weapons, radar systems, and aerospace technologies. Given their strategic importance, REEs are often termed “technology-critical elements” or “industrial vitamins”, because of their fundamental role in sustaining modern industrial processes [10]. In addition, REEs are being explored and used in the field of biomedicine, particularly as contrast and theranostic REE-based pharmaceutics [11].

REEs are naturally present in over 270 different minerals, with primary deposits typically occurring in bastnasite, monazite, and xenotime, which account for roughly 95% of REE resources globally [12]. These minerals are predominantly found in igneous and sedimentary rock formations, and their REE content is influenced by factors such as the geochemical conditions and mineral genesis, which have varying affinities for different REE subgroups. Bastnasite, for example, is rich in light LREEs, while xenotime predominantly contains HREEs, influencing their geological distribution and extraction methods [12]. In response to the demand for REEs, today these elements are sourced globally, with significant deposits in China, the USA, Brazil, and Australia. China holds the largest known reserves and dominates global production [7].

**Figure 2:**
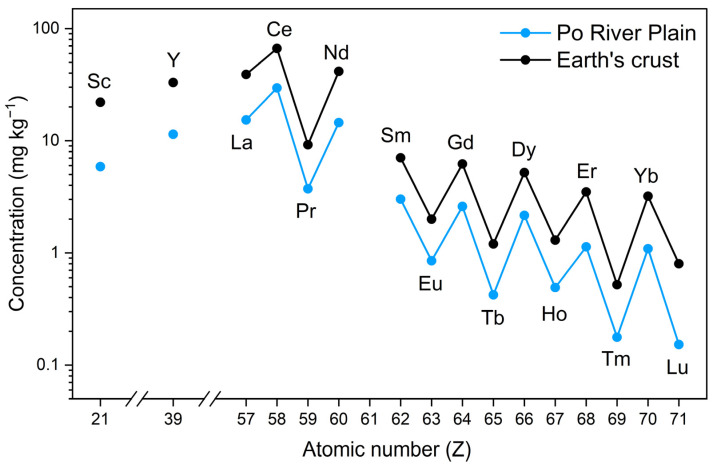
Comparison of average REE concentrations in the Earth’s crust (black line) and Po River Plain (blue line), Italy. Values are expressed in mg kg^−1^ on a logarithmic scale, as a function of the atomic number (Z). The values show consistently lower REE concentrations in the Po River Plain compared to the Earth’s crust, owing to sedimentary processes and geochemical fractionation occurring in fluvial environments. Nevertheless, the characteristic “zig-zag” pattern of REE abundances, explained by the Oddo–Harkins rule [16], is preserved, reflecting the greater stability and abundance of elements with an even Z compared to those with an odd Z. The Earth crust values are from [17]; the Po River Plain data are provided by Renzo Tassinari, University of Ferrara. The graph was created with OriginPro 2025 (OriginLab Corporation, Northampton, MA, USA).

The natural concentration of REEs in soils is primarily influenced by the composition of the parent rock materials, which gradually release REEs during weathering processes [10,13,14,15]. In particular, REEs tend to follow the Oddo–Harkins rule, where elements with even atomic numbers are naturally more abundant than those with odd atomic numbers (Figure 2). This rule influences REE distribution patterns in soil and complicates the analysis and interpretation of their behavior in various environments. Several soil characteristics, such as the presence of carbonates and phosphates, pH, clay mineralogy, organic matter content, bacterial and fungal microflora, and plant roots, also affect the mobility and bioavailability of REEs [15], as will be dealt with in Section 2. Different anthropogenic activities further contribute to REE levels in soils. However, the long-term effects of REE enrichments on soil health and the ecosystem remain uncertain. With the increasing use of REEs, understanding their environmental fate and ecological implications is crucial. Elevated REE concentrations in soils, particularly in proximity to industrial sites or in regions with intensive agriculture, could pose risks to soil organisms, plants, and potentially human health through bioaccumulation. As such, there is a growing need for comprehensive studies not only on REE bioavailability, benefits, and toxicity, but also on their specific and non-specific interactions with the structure and metabolism of plants. In fact, the presence of REEs in plants as primary producers depends on the soil-to-plant continuity of such xenobiotics, subsequently passed to consumer organisms, humans included. The exploration of sustainable practices, such as the development of REE recycling processes, is also gaining momentum, in order to reduce our dependence on primary mining and mitigate the environmental impacts of these xenobiotics [9].

## 2. Basic Chemical Information About REEs

The primary oxidation number of all REEs is +3; however, europium (Eu) can also easily be divalent and Ce tetravalent. REEs are characterized by a pronounced ionic nature, with their interactions dominated by electrostatic forces rather than covalent bonding. This behavior arises from their high positive charge and the minimal contribution of their 4*f* electrons to bonding, as these electrons are shielded by outer electron shells and remain largely inert. Consequently, REEs exhibit a strong preference for anionic ligands with highly electronegative donor atoms [18]. Specifically, acting as hard Lewis acids, owing to their small ionic size, high charge density, and low polarizability, REEs form the most stable complexes with oxygen and fluorine, as small, highly electronegative, and non-polarizable hard Lewis bases [19]. A reader interested in the specificity of the redox and coordination chemistry of REEs can refer to reviews on this specific subject [11,18,20,21].

**Figure 3:**
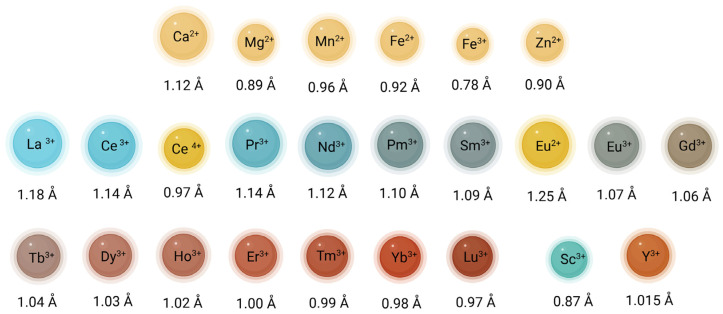
Ionic radii of REEs and selected elements with key roles in plant biological processes. The comparison emphasizes the possibility that some REEs may substitute for certain micronutrients with a similar ionic size. The close dimensional similarity between Ca^2^⁺ and many REEs explains why these elements are very likely to replace Ca^2^⁺ in plants. Similarly, specific REEs may be absorbed based on their similarity in size to other essential elements like Mg, Fe, Zn, and Mn, which play vital roles in plant metabolism. The oxidation state significantly influences the ionic size of Ce and Eu, thereby affecting their potential to substitute for Ca^2^⁺ or other essential elements. Furthermore, the graph highlights the “lanthanide contraction”, i.e., the progressive decrease in ionic radius across the lanthanide series, attributed to the poor shielding effect of *f*-electrons. All the ionic radii data were obtained from [22]. The image was built with BioRender (https://www.biorender.com/, BioRender Inc., Toronto, ON, Canada).

It is common to classify the REEs into two groups, based on their atomic numbers (Figure 1). The light REEs (LREEs) include the first seven lanthanides, La, Ce, praseodymium (Pr), neodymium (Nd), Pm, and samarium (Sm), along with Sc. The last eight lanthanides are referred to as heavy REEs (HREEs): gadolinium (Gd), terbium (Tb), dysprosium (Dy), holmium (Ho), erbium (Er), thulium (Tm), ytterbium (Yb), and Lu. Though lighter than the lanthanides, Y has a reactivity similar to HREEs [18]. Eu lies between the LREEs and the HREEs. The ionic radius decreases from La^3+^ to Lu^3+^, representing the “lanthanide contraction”, which follows the increasingly positive charge of the nuclear atom. The REEs ionic radii are reported in Figure 3, in comparison with those of elements used by plants as micronutrients. The similarity of their ionic radius with that of Ca^2+^ is particularly evident for the LREEs. Other essential cationic micronutrients are considerably smaller.

## 3. Geochemistry of REEs in Soils

### 3.1. Influence of Parent Rocks

**Figure 4:**
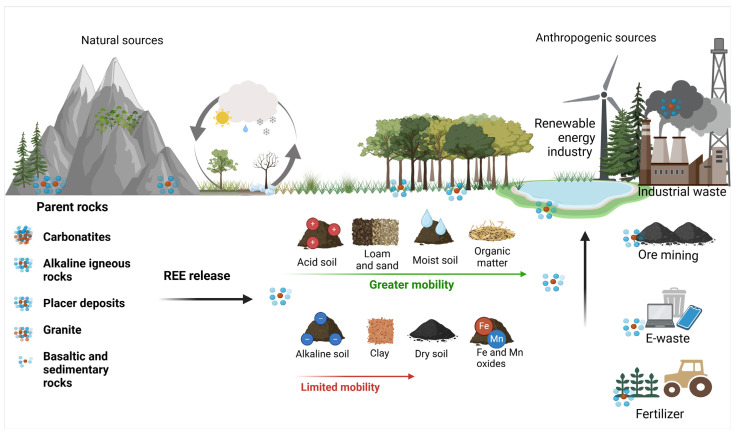
REEs originate from natural and anthropogenic sources, undergoing various processes that influence their release, mobility, and environmental impact. Natural sources include a variety of parent rocks, from which weathering processes driven by precipitation, temperature variations, and erosion facilitate the release of REEs into the environment. Once released, their mobility in soils depends on several physicochemical conditions, which also influence their bioavailability. Anthropogenic activities significantly contribute to REE dispersion, primarily through ore mining, industrial waste discharge, electronic waste (E-waste) disposal, and fertilizer application. These activities lead to the accumulation of REEs in terrestrial and aquatic *eco*systems, potentially disrupting natural biogeochemical cycles and posing environmental risks. The image was built using BioRender (BioRender Inc., Toronto, ON, Canada).

The geochemistry of REEs in soils is profoundly influenced by the type of parent rock, which determines their concentrations, mobility, and distribution patterns (Figure 4). The parent materials play a vital role in the enrichment or depletion of REEs, shaped by their mineralogical composition and geochemical behavior during rock formation and subsequent weathering [12,14,23]. Igneous felsic rocks, such as granites, are typically enriched in LREEs, because of the geochemical processes that occur during their formation. Magmatic differentiation causes LREEs to preferentially partition into the residual melt, resulting in their accumulation in late-stage felsic products. This enrichment is particularly evident in the continental crust, where granitic rocks dominate, exhibiting a strong LREE signature. In contrast, mafic rocks like basalts, which crystallize at higher temperatures, display lower overall REE concentrations, but a more balanced distribution between LREEs and HREEs. This balance is due to HREE incorporation into early forming minerals, such as garnet and clinopyroxene, during mafic magma crystallization [12,24,25].

Sedimentary rocks add another dimension to REE geochemistry. Argillaceous sediments, rich in clay minerals, often contain elevated REE concentrations, owing to the affinity of REEs to clays and their ability to form complexes with organic and inorganic ligands. These sediments act as efficient REE reservoirs, thanks to their high surface area and ion-exchange capacity [26,27,28]. Carbonate rocks, such as limestones and dolomites, typically exhibit lower REE concentrations, because carbonate minerals are less compatible with REEs. However, they may inherit REE patterns from precursor materials or undergo diagenetic changes that influence their REE content [29,30]. Sandstones, primarily composed of quartz, generally show the lowest REE concentrations, which is due to the removal of fine-grained, clay-rich materials during their formation [31,32].

### 3.2. Geochemical Processes Affecting REE Mobility and Bioavailability in Soil

The distribution of REEs, comprising the lanthanide series, in soils is an important geochemical marker for soil–plant systems and is influenced by the parent material composition and environmental conditions, which in turn affect plant uptake (Figure 4). Naturally, REEs enter soils through the weathering of REE-bearing minerals in the parent rock material. Common primary sources include carbonatites, alkaline igneous rocks, and placer deposits containing minerals, such as bastnasite, monazite, and xenotime. Carbonatites and alkaline rocks represent some of the world’s richest sources of REEs, owing to their unique mineral composition and high REE content. As these rocks weather, REEs are gradually released from their primary minerals into the soil environment, where they can either remain bound within secondary minerals or they interact with clay minerals, oxides, and organic matter. While the absolute REE content in soils derived from different rock types varies widely, with granitic soils typically richer in REEs than basaltic or sedimentary soils [33,34], additional differentiation can also occur. Particularly, felsic rocks often release HREEs preferentially, relative to LREEs, during weathering, because LREE-bearing accessory minerals, like monazite and allanite, are more resistant to breakdown [35] (Figure 4).

The multiple, complex interactions between parent rock types, mineralogy, and weathering processes underscore the critical role of geology in shaping the REE pattern of soils, and, in particular, are the first fundamental piece of knowledge in order to understand how REEs enter plants and, consequently, the biosphere, as xenobiotics. A further advancement in terms of understanding the relationship of REEs to plants requires us to consider other crucial physical–chemical properties of soils, particularly local environmental factors.

The behavior of REEs in soils is intricately governed by the soil texture and presence of secondary minerals. Fine-textured soils with a high proportion of clay minerals and secondary phases can provide abundant adsorption sites for REEs (Figure 4). The high surface area and the presence of negatively charged functional groups make iron (Fe) and manganese (Mn) oxides potent sorbents that strongly bind REEs through surface complexation, leading to the retention of REEs [10]. These oxides not only sequester REEs, but also create chemical environments that promote fractionation among individual REEs. HREEs, with their smaller ionic radii and higher charge density (Figure 3), tend to exhibit stronger interactions with these minerals compared to LREEs. Moreover, HREEs are generally less mobile than LREEs, because of their stronger association with more resistant mineral phases, such as zircon. Interestingly, weathering and sorption can act in opposite directions, with the preferential release of HREEs from felsic rocks being counteracted by their stronger retention of HREEs on mineral surfaces. Altogether, this differential geochemistry related to the atomic mass of REEs results in distinct REE distribution and mobility patterns across soil horizons [36,37].

In addition to the cation exchange capacity of soils, the mobility and retention of REEs are further influenced by the water availability, organic matter content, and overall redox state (Figure 4) [38,39]. In arid and semi-arid regions, where organic matter and water are limited, REEs tend to remain immobilized. This is due to the predominance of stable mineral phases and the reduced intensity of leaching processes. The restricted movement of water prevents significant REE dissolution and transportation, resulting in their retention in forms such as phosphates, oxides, and carbonates, which exhibit low solubility in these conditions [23,34]. The oxidative conditions of arid soils further stabilize REEs by limiting processes such as Ce^3+^ oxidation to the less mobile Ce^4+^, which exhibits unique behavior, forming insoluble CeO_2_ and contributing to Ce immobilization and distinctive REE fractionation in soils [40].

In contrast, higher levels of organic matter, increased water fluxes, and fluctuating redox conditions of humid soils foster greater REE mobility and bioavailability. The presence of organic acids derived from decomposing organic matter promotes REE complexation, particularly with humic and fulvic acids. These soluble organo-metallic complexes enhance the leaching of REEs from soil matrices, enabling their redistribution through the soil profile, and provide extended availability to the microflora and the plant roots exploring the soil space, collectively influencing the ecological dynamics [41,42]. The acidic soil conditions often prevalent in humid regions, and also actively determined by plant roots (see Section 4.1), contribute to the dissolution of REE-bearing minerals [43].

REEs exhibit significant interactions with sulfate minerals, particularly within the acidic environment of acid sulfate soils [44]. These interactions are primarily driven by geochemical processes, such as sulfide oxidation, which creates acidic conditions favoring REE mobilization. Studies reveal a strong association of REEs with secondary Fe(III) oxyhydroxysulfates like jarosite and schwertmannite, facilitated by surface adsorption and structural incorporation [45]. This often leads to the fractionation of REEs, with HREEs exhibiting stronger binding affinities and preferential retention compared to LREEs. Shale-normalized REE patterns frequently display enrichment in REEs with intermediate Z (Sm, Eu, Gd), attributed to their affinity for carbonate and sulfate minerals [44,45,46]. The presence of organic matter and colloids further complicates REE behavior, influencing their bioavailability and transportation [10,34]. Geochemical investigations in Swedish and Finnish coastal plains highlight the significant influence of drainage and the climate on REE distributions, with drained soils exhibiting accelerated REE release because of increased sulfide oxidation [46]. These findings emphasize the dynamic nature of REE behavior in sulfate-rich environments, where sulfate minerals act as both sources and sinks for these critical elements [33].

Additionally, humid soils are more prone than arid soils to redox oscillations, particularly the reducing environment in waterlogged or water-saturated soils prevents the oxidation of Ce^3+^ and Eu^2+^ [10]. Collectively, humid, acidic, and reducing conditions generally increase REE mobility and availability for uptake by plants, while neutral-to-alkaline soils tend to immobilize REEs within stable mineral complexes [47,48,49].

In addition to natural sources, anthropogenic activities have increasingly introduced REEs into soils, particularly in agricultural and industrial regions [50] (Figure 4). Phosphate fertilizers are a significant source of REEs in agricultural soils: phosphate rocks, which are commonly used in fertilizer production to enhance agricultural productivity, often contain considerable REE concentrations [7]. However, REE content in fertilizers can vary widely depending on the geological origin of the phosphates and the processing methods used, leading to a range of potential REE inputs in agricultural soils [7]. Soils historically fertilized with phosphates are expected to accumulate significant amounts of REEs, particularly in the surface horizons. A 2021 study by Jiménez-Ballesta et al. on vineyard soils in the province of Ciudad Real (central Spain), shows that the spatial distribution of REEs as a product of parent rock degradation is complicated by localized anomalies generated by the anthropogenic input of phosphate fertilizers [51]. Other agricultural practices can influence the abundance of REEs in soils, for example, Punturo et al., in 2018 [52], observed a positive Eu anomaly in the soils of the Hyblean Plateau, in southeastern Sicily (Italy), which they attributed to the propensity of Eu^2^⁺ to substitute for Ca^2^⁺ in silicate structures within the soil minerals, a process further enhanced by the common agricultural practice of mixing “red soil” with carbonate terrains to improve soil quality [52].

Mining activities, metallurgical processes, industrial emissions, and wastewater discharge are also known to contribute to REE enrichment in soils, especially in areas close to industrial centers or mines (Figure 4) [15]. Urban areas and regions near industrial sites often display elevated REE levels due to localized anthropogenic sources [23,50,53]. As will be briefly mentioned in Section 5, a significant source of REEs in soils is also the deliberate use of REEs in agriculture as enhancers of crop performance.

The cumulative effect of these interactions shapes the geochemical cycling of REEs in soils, determining their long-term fate and environmental implications. By immobilizing REEs through adsorption and complexation, soil components act as both reservoirs and regulators, ensuring that REEs remain largely confined within specific soil compartments, thus minimizing their bioavailability and the potential ecological risks. The increasing introduction of REEs into soils through human activity highlights the need for further research on their environmental behavior, potential bioaccumulation, and the long-term ecological impacts [23,33,34,51].

## 4. Absorption and Fractionation of REEs in Plants

**Figure 5:**
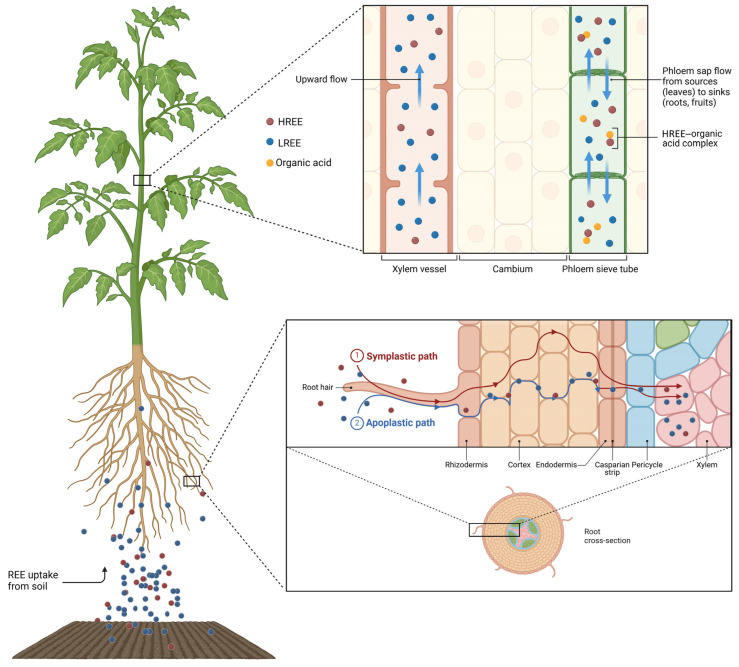
Uptake and transportation pathways involving REEs in plants, starting from the soil. The lower panel details the absorption pathways and the role of the Casparian strip in regulating the entry of REEs into the vascular system. REEs are initially absorbed mainly through the apoplastic pathway, diffusing through cell walls and extracellular spaces. At the level of the Casparian strip, a selective process occurs, allowing a higher proportion of LREEs to cross compared to HREEs. Beyond the Casparian strip, REEs continue through the symplastic pathway, moving through the cytoplasm via plasmodesmata, reaching the xylem vessels for upward flow toward the aerial parts of the plant (upper panel). In the phloem sieve tubes, REEs are redistributed from sources (e.g., leaves) to sinks (e.g., roots and fruits) as REE–organic acid complexes, facilitating their mobility. The binding to organic acids promotes relative HREE enrichment in the phloem sap. The image was built using BioRender (BioRender Inc., Toronto, ON, Canada).

The mobility and distribution of REEs in plant tissues depend on the mechanisms by which plants absorb REEs from the soil and transport them. The processes of fractionation between LREEs and HREEs during translocation are examined here, shedding light on the role of cellular barriers and transport systems. Figure 5 provides a comprehensive visual representation of REE transportation in plants, illustrating their movement from the rhizosphere into the xylem. It also highlights the differential transportation and redistribution of REEs, emphasizing the role of organic acid complexes in facilitating their mobility through the phloem and their accumulation. We also explore the role of root exudates, microbial interactions, and symbiotic relationships in enhancing REE bioavailability and managing their potential toxicity.

### 4.1. What Happens Around the Roots: Events in the Rhizosphere

The availability of REEs for plant uptake is influenced by several factors in the rhizosphere, including the pH, organic matter, REE speciation in soil fractions, and the formation of complexes with minerals like phosphates or sulfates [54]. Generally, at high pH levels, REEs tend to form insoluble complexes, limiting their uptake by plants. Conversely, acidic soils enhance REE bioavailability by promoting the dissolution of REE-bearing minerals [47,55].

The coordination chemistry of REEs plays a key role in their availability for absorption by plant roots, especially considering the affinity of these elements for oxygen donor ligands [56]. For this reason, organic matter, particularly humic acids, form complexes with REEs, affecting their speciation, binding, and mobility in soils. Additionally, the granulometric soil structure, cation exchange capacity, and microbial diversity indirectly impact REE bioavailability [57,58]. As reported in Section 3.2, their availability is also influenced by their complexation with clay minerals, phosphates, or sulfates in the soil solution. Furthermore, the amount and lability of Fe–Mn hydroxy compounds are critical in the complexation of REEs [1]. Accordingly, in *Persea americana*, a positive correlation was observed between REE uptake and Mn and Fe accumulation in the roots [59].

Plants themselves influence REE bioavailability through their root exudates, which form REE–organic complexes, reducing the toxicity of REEs and, at the same time, facilitating their absorption [60]. These exudates can include carbohydrates, amino acids, enzymes, indole compounds, or organic acids [61]. Phytosiderophores, which are generally recognized for their role in Fe uptake, may also act as carriers for REEs [62], as is also known for toxic metals, generally enhancing their release [63]. This hypothesis could be supported by the formation of a Eu–nicotianamine complex, which was obtained in vitro [64]. Accordingly, the phytosiderophore, desferrioxamine B (DFOB), has been shown to significantly increase the mobility of REEs in soil and their uptake into the shoots of *Phalaris arundinacea* [55]. Additionally, naturally occurring rhizosphere bacteria, such as *Arthrobacter oxydans* and *Kocuria rosea*, release various chemical compounds resembling desferrioxamine, bacillibactin, and surfactin. These compounds effectively mobilize REEs and enhance their bioavailability in the soil [65]. Moreover, in response to environmental factors, root-associated microorganisms can form beneficial microbial consortia to alleviate the effects of combined contamination. Examples of this include *Bradyrhizobium*, *Rhizobium* and *Candida koribacter*, which act as anti-REE-stress microorganisms, thereby promoting plant growth under stress conditions [66].

REE bioavailability can also be affected by arbuscular mycorrhizal fungi (AMF). Some research suggests that AMF can alter the structure of microbial communities in the rhizosphere, enhancing the plant’s tolerance to REE stress [64]. Numerous studies have demonstrated that under abiotic stress conditions, AMF enhance nutrient uptake, stimulate plant growth, upregulate the synthesis of phytochelatin synthetases (PCs) and glutathione (GSH) for metal chelation, activate antioxidative enzymes, and reinforce non-enzymatic defense systems [67,68,69,70,71]. For instance, maize seedlings under La stress demonstrated enhanced resistance when in symbiosis with *Claroideoglomus etunicatum*. The underlying mechanisms involve the upregulation of AUX/IAA genes, which are crucial in auxin signal transduction, and the enhanced expression of ABC transporters, Nramp6, as well as genes associated with vacuole and vesicle compartmentalization [72]. In general, the role of AMF in REE mobilization and transfer to plants is not yet fully understood and requires further investigation [73,74].

### 4.2. Root Surface Interactions

The epidermis of plant roots, known as the rhizodermis, plays a critical role in controlling the availability and mobility of REEs. The rhizodermis, the outermost cell layer in the actively absorbing zone of the root, is characterized by root hairs, which grow between the soil particles and increase the root absorption surface. REEs tend to precipitate at the rhizodermis, owing to interactions with phosphate and hydroxide ions in the rhizosphere. This precipitation forms insoluble compounds, such as LaPO_4_ and CePO_4_, limiting REE absorption by the plant [73]. Additionally, REEs are highly prone to bind with carboxyl groups of pectins in the cell wall: the positive and negative charges are attracted to each other, leading to selective adsorption of REE ions by the cell wall, with strong affinity [73]. For instance, in the fern *Pronephrium simplex*, REEs are associated with the cell wall, very likely substituting for Ca^2+^ ions [75]. Furthermore, REE–phosphate precipitates have been observed in the root apoplast of *Zea mays*, potentially contributing to REE immobilization [76]. Instead, although organic acids, such as citrate, can increase REE solubility and facilitate their uptake, the presence of high phosphate concentrations in the soil often overwhelms this effect, leading to further precipitation and reduced mobility [77]. This observation suggests that in the cell wall, REE^3+^ cations could be trapped between the carboxyl groups of the pectins and the free phosphate, in analogy to Fe^3+^ chelation in –COO-Fe-PO_4_ complexes [78]. In *Oryza sativa*, it was shown that the plant cell regulates the methylesterification level of the carboxyl groups in galacturonic acid residues to fine tune phosphate availability [79]; under low phosphate conditions, the remobilization of phosphate allowed by pectin demethylation could result in a higher level of mobility of REEs in the cell wall matrix and promote their uptake.

### 4.3. Upon Entering the Root

Despite REE adsorption and chelation by organic acids or precipitation on the root surface, a fraction of REEs can nevertheless pass through the pores in the rhizodermal cell walls and move along the apoplastic pathway. The flow of REEs in the extracellular matrix is finally blocked by the Casparian strip at the endodermis, which forces them into the symplastic pathway through the plasma membrane (Figure 5) [1,55,80]. In fact, the Casparian strip is an ion selective barrier within the plant root, formed by the hydrophobic impregnation of lignin in the anticlinal cell walls of the endodermis [81,82], preventing the uncontrolled flow of water and solutes from the root cortex to the vessels in the central cylinder [83]. In the 1970s, the Casparian strip was identified by Nagahashi et al. as a barrier to the movement of La^3+^ in *Zea mays* roots [84]. La deposits were observed exclusively in the cell walls and on the outer surface of the plasma membrane of epidermal, cortical, and endodermal cells, up to the Casparian strip, but were completely absent from the stele. This finding was corroborated by numerous studies, which provided strong evidence that the Casparian strip prevents the movement of REEs [82,83]. More recent studies further support the idea that the Casparian strip blocks, or at least modulates, REE uptake in the roots and their subsequent transfer to the aerial parts of the plant, confirming the efficient filtering capacity of the endodermis [81,82,83,84,85,86,87].

Through the symplastic pathway, REEs are actively transported into the protoplasts via membrane carriers, such as NRAMP and ABC transporters [72]. In a study by Zhang et al. where *Zea mays* plants were tested under La stress conditions, the differentially expressed genes annotated as ABC transporters accounted for 17% of all DEG transporters. All nine genes of the G subfamily were upregulated, with two of them showing a ca. 4–6-fold expression increase. Moreover, two differentially expressed proteins among the ABC transporters, including ABC transporter F family member 1 and ABC transporter G family member 37, were also upregulated [72]. The G subfamily of ABC transporters, which includes the largest number of transporters, supports plant growth by enhancing its resistance to abiotic stress [88]. Therefore, the uptake of REEs shares similar transport mechanisms with other elements, especially Ca^2+^, often competing for the same binding sites [34,89,90,91]. For instance, LREEs enter the roots of *Phytolacca americana* via Ca^2^⁺ channels [92], and competition between La^3^⁺ or Ce^3^⁺ and Ca^2^⁺/Mg^2^⁺ has been observed in *Triticum aestivum* [93]. Some studies have highlighted that Al^3+^ transporters may also be involved in the uptake of REEs, as demonstrated for Y^3+^ [94]. In the REE hyperaccumulator fern, Dicranopteris linearis, the NRAMP REE Transporter 1 (NREET1) facilitates REE uptake from the root cell wall to the cytoplasm [95]. Interestingly, while NRAMP transporters are normally used by plants for divalent cations (Fe^2+^, Mn^2+^, Zn^2+^), this is not the case for NREET1.

**Figure 6:**
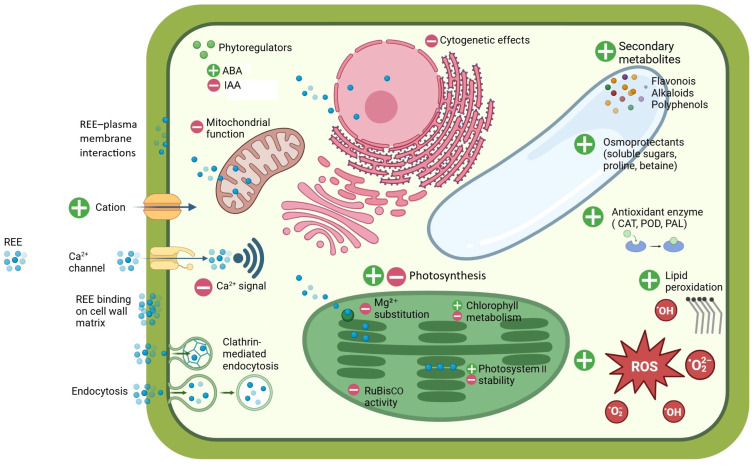
REEs bind extensively to the cell wall matrix, mainly pectins, and facilitate endocytosis, either clathrin-mediated or not. REEs also interact with the plasma membrane, facilitating cation uptake and influencing Ca^2^⁺ signalling. Inside the protoplast, REEs exhibit both positive and negative effects on various functions, based on their concentration (hormetic effect). The mitochondrial functionality can be impaired, while chloroplasts experience mixed effects on photosynthesis, including possible Mg^2^⁺ substitution in chlorophyll, reduced RuBisCO activity, and diverse impacts on chlorophyll metabolism and PSII stability. Note that the abundant negative charges at the thylakoid surface in the grana partitions offer many chances for ionic REE interactions. In the cytosol, REEs influence the production of phytohormones and reactive oxygen species (ROS). ROS lead to lipid peroxidation and stimulate the production of secondary metabolites, osmoprotectants, and antioxidant enzymes, playing a protective role against oxidative stress. At the nuclear level, REEs can induce negative cytogenetic effects. The image was built using BioRender (BioRender Inc., Toronto, ON, Canada).

As an interesting alternative to the opportunistic use of cell membrane transporters, research has highlighted the role of endocytosis in the uptake of REEs. REEs can anchor to the plasma membrane in the form of nanoscale particles, initiating endocytosis and, thereby, increasing their uptake into plant cells [96]. In particular, REEs interact with glycoproteins found at the interface between the cell wall and the plasma membrane, such as FLA17, a secretory FASCICLIN-like arabinogalactan protein (FLA) localized on the plasma membrane. Tb^3^⁺ binding to FLA17 modifies the interactions between the extracellular matrix and the protoplast: Tb-FLA17 contacts the intracellular protein, articulin (AP2), and forms a kind of trans-membrane Tb-FLA17-AP2 complex receptor, which activates clathrin-mediated endocytosis and leads to the internalization of the REE (Figure 6) [97].

At the outer surface of the plasma membrane, REEs may also cross-link with lipids, changing the membrane fluidity, pore size, and electrical properties, further influencing ion transport and membrane-bound enzyme functions [98,99,100]. The La-induced enhancement of endocytosis in the roots has recently been shown in *Solanum nigrum*, upon the foliar application of 10 μM La^3^⁺ [101]. This process was related to changes in DNA methylation, as mediated by the expression of genes encoding DNA methylases and demethylases. The key genes include CMT3, DRM2, and DNMT2 after 12 h of exposure and expand to MET1, CMT1, CMT2, CMT3, DRM2, DNMT2, ROS1, DME, DML2, DML5a, and DML5b after 24 h [99]. Interestingly, increased endocytosis further stimulates the expression of these genes, creating a feedback loop that reinforces the uptake process [102].

The endocytic uptake mechanism of REEs has also been observed in leaves [96,97]. In leaves, REEs bind to arabinogalactan proteins (AGPs) on the outer surface of the plasma membrane, forming Lewis acid base complexes that are then distributed across the membrane and activate endocytosis [97]. The facilitation of REE uptake by endocytosis is also supported by a well-known interference of these elements with calmodulin (CaM), a critical Ca^2^⁺ sensor involved in numerous cellular processes, including membrane remodeling and endocytic vesicle formation [103]. The REE–CaM interaction is discussed in Section 5.2.

The REE–plasma membrane interactions can also result in the enhanced uptake of other elements, although the precise mechanisms remain unclear [89,104,105]. For examples, the treatment of *Nymphoides peltata* with Y^3^⁺ of 1–5 mg L^−1^ for 4 days increased the levels of minerals such as Mg, Ca, Fe, Mn, and molybdenum (Mo) [106]. Similarly, Wang et al. reported that optimal Ce^3^⁺ concentrations (20 mg L^−1^) increased the level of K, Mg, Ca, Cu, Fe, Mn, and other minerals in *Armoracia rusticana* [107]. In *Zea mays*, with La^3^⁺ and Ce^3^⁺ concentrations of up to 0.9 mM, the Mg and K uptake increased, while the Ca, Mn, and Zn levels decreased [90]. Therefore, the effect of REEs on the mineral content of plants varies based on the plant species and soil conditions. For example, in *Triticum aestivum*, *Brassica napus*, and *Cicer arietinum*, REE accumulation increased under P-deficient conditions. In contrast, under similar conditions, in *Pisum sativum*, *Lupinus albus*, and *L. cosentinii*, the REE uptake decreased [108].

Once inside the cell, REEs can be bound by intracellular chelators, such as organic acids (e.g., citric or malic acid) or phytosiderophores. These compounds form complexes with REEs, reducing their toxicity and facilitating their transportation through the cytoplasm. Chelation is crucial for preventing cellular damage due to the accumulation of non-essential metals like REEs, which can interfere with cellular processes [109,110].

### 4.4. The Route for REE Translocation to the Shoot and Their Fractionation

After REEs are absorbed and processed in root cells, those overcoming the endodermis are transferred into the xylem vessels for transportation to the aerial parts of the plant (Figure 5). Xylem transport of REEs is mediated by water flow and driven by leaf transpiration. During this process, REEs may interact with organic acids or chelators, such as citrate or malate, which can help reduce their toxicity and enhance their solubility, making them easier to transport [111]. For instance, in *Saxifraga paniculata*, Y was found to co-localize with Al, Fe, and Ce in the roots, stems, and leaves, and the authors propose that citrate might help bind and transport these elements within the plant [94]. REEs can also undergo fractionation, with LREEs tending to move more freely than HREEs because of their different chemical properties, including their varying solubility and affinity for chelators [58,77,112]. Selective transport and chelation help regulate the concentration of REEs within different plant tissues and prevent their potential toxicity. As a result, while REEs primarily accumulate in the roots, they can also be translocated to the shoots, with the efficiency depending on the plant species and environmental conditions [47,48,55,67,68].

Although REEs are transported to the aerial parts of the plant via the xylem, they can be subsequently redistributed to the plant organs, roots included, through the phloem [113]. Different from the root-to-leaf ascensional xylem flow, the phloem sap moves inside the sieve tubes from the leaves (sources) to the sink plant tissues, according to the needs in regard to the photosynthates of the latter. For example, phloem transportation can distribute nutrients from mature to younger, developing leaves. In general, phloem transportation is much more selective than xylem transportation and involves active mechanisms [114]. Phloem sap, rich in organic compounds, facilitates metal complexation and long-distance transportation, with REEs binding to low-molecular-weight organic acids, such as citric, malic, and oxalic acids [115,116]. Additionally, phloem transportation is thought to play a key role in the differential accumulation of HREEs in the aerial organs of some plants. Indeed, Guo et al. demonstrated the bi-directional translocation of REEs through the phloem in *Phytolacca americana*. In their study, 86% of REEs were transported back from mature leaves to the roots, while only 14% moved upward to younger leaves. The presence of oxalic acid in phloem exudates was found to be critical for the long-distance transportation of HREEs [117]. These observations suggest that, while the REE concentration is universally decreasing from the soil to the roots to the leaves, their actual distribution in the various plant organs may not follow a simple model.

The distribution of trace elements in plant tissues is the result of elemental absorption selectivity at the root level and, subsequently, of the differential attitude to locate them to a certain plant compartment, for e.g., leaves, fruits, and seeds, resulting in so-called elemental fractionation. The distribution of REEs in plants is primarily influenced by the presence of apoplastic barriers; they play a crucial role in restricting the movement of REEs, leading to an accumulation pattern that typically follows the order: roots > stems > leaves > flowers > fruits > seeds. REE uptake can also occur through foliar application, in which case the apoplastic barriers still hinder the translocation of REEs to other plant parts. However, the distribution pattern shifts, with the highest concentrations observed in the leaves, followed by the stems, roots, flowers, fruits, and seeds [34].

In most cases, REE concentrations in plant tissues are roughly proportional to their content in the soil, typically reflecting the geological characteristics of the surrounding environment [13,118]. However, REEs also undergo fractionation within plants, in relation to the plant species, physiological and anatomical differences among plants, as well as the environmental conditions and the specific element in question [92,117,119]. As already mentioned, variations in the cell wall composition, particularly in polysaccharides, play a critical role in REE interactions [120,121,122]. Additionally, molecules, such as organic acids or REE-binding peptides produced by different plant families, may also contribute to the patterns of elemental distribution. Atomic weight is another potential factor in REE fractionation, based on evidence mainly obtained from REE accumulators. In this respect, interestingly, the distinct REE fractionation patterns between plant groups seem to be conserved, with ferns preferentially accumulating LREEs and angiosperms preferentially accumulating HREEs [19]. The preferential translocation of HREEs to the shoots has been observed in *Phytolacca americana* grown under both hydroponic conditions and in natural REE-mining areas [123]. In non-accumulating species, a higher root-to-shoot transfer of HREEs compared to LREEs has been reported in various angiosperms, including *Triticum aestivum*, *Glycine max*, and *Oryza sativa* [124,125]. The HREEs vs. LREEs fractionation may be explained by the production of specific compounds with distinct REE-chelating properties. For example, the REE-accumulating fern, *Dicranopteris dichotoma*, produces a specific LREE-binding peptide, which may play an important role in their hyperaccumulation. Probably LREE-binding peptides may be engaged in the detoxification and homeostasis of LREEs, suggesting that the LREE-binding peptide is less toxic to cellular plant metabolism than free metal ions [126]. On the contrary, HREE enrichment in *Phytolacca americana* is associated with the long-distance transportation of HREE–citrate complexes [117]. It is not known whether, or to what extent, the conclusions obtained with metal-accumulator plants is valid in non-accumulator species. Moreover, it is conceivable that the fractionation of REEs may have significant variability even among varieties of the same species, or crop cultivars, albeit this aspect has not yet been addressed by dedicated fundamental research.

In addition to the dichotomy of LREEs–HREEs, there is a specific REE, Eu, which frequently exhibits a higher absorption rate compared to the others. While the positive or negative Eu anomaly known in regard to rocks and soils (see Section 3) can be reflected in plant organs [48,49,127], relative Eu enrichment was observed in the aerial parts of several species, such as wheat [125], spruce, beech [81], and grapevine [128]. Various hypotheses have been proposed to explain the origin of these anomalies. One hypothesis suggests that the close similarity in the ionic radii between Eu^3^⁺ (1.07 Å) and Ca^2^⁺ (1.12 Å) leads to the substitution of Ca by Eu in plants growing in Ca-deficient soils [125]. An alternative hypothesis, proposed by Ding et al., involves the precipitation of Eu-rich phosphates within plant tissues [129]. Krzciuck et al. proposed that the Eu anomalies observed in *Juncus effusus* are the result of redox fluctuations in the rhizosphere of this wetland plant species [130]. During photosynthetic activity, oxygen is released through aerenchyma into the roots, where it is consumed by aerobic microorganisms. This consumption leads to a significant drop in the plant’s redox potential during the night. Under these low redox conditions, Eu^3^⁺ is reduced to Eu^2^⁺ [125]. In its reduced form, Eu exhibits higher bioavailability and mobility compared to Eu^3^⁺ and other REEs [131].

## 5. Impacts of REEs on Plant Processes

### 5.1. Hormetic Action of REEs on Plants

REEs have been increasingly recognized for their beneficial effects in agriculture, where they can be applied as micro-fertilizers to enhance plant growth and development. For instance, in wheat and soybean crops, they have been successfully used to increase yields by up to 15% [132]. These positive effects are largely attributed to the ability of REEs to act on various physiological and biochemical pathways, including the regulation of nutrient absorption and the activity of key enzymes, as well as increasing the chlorophyll content and enhancing the photosynthetic efficiency of plants, which leads to greater biomass production [13,87,133]. The use of REEs in agriculture is outside the main scope of this review, and, in particular, a reader can refer to the comprehensive analyses reported by Tommasi et al. and Kastori et al. [134,135] in this regard. However, interestingly, the extensive agronomic use of REEs remains limited in practice to China, although the commercial use of REE-enriched fertilizers is also documented in the USA and the UK [136,137]. Nevertheless, the use of REEs as fertilizers is a cause of relevant environmental concerns. The application of REEs in Chinese agriculture since the 1980s is one of the most probable causes of the enrichment of REEs in rainwater [138] and, moreover, exogenous REE input in soil enhances the emission of the greenhouse gas, N_2_O, because of its interactions with soil microbial flora [139]. In fact, modern agriculture is one of the human activities deemed responsible for the “cryptic entry” of REEs into natural resources and the environment, with toxic effects for life on Earth [140].

Despite the fact that most of the research has focused only on La and Ce, it can be generalized that REEs have a biphasic action in regard to plants, also known as the “hormetic effect”: at low concentrations, they exhibit positive actions on plant performance, while above a certain threshold, they bring about a wide range of toxic effects [141,142]. Until recently, information on the metabolic and developmental effects of REEs on plants was mostly empirical, and research papers did not delve into the underlying mechanisms, especially with regard to the beneficial effects. A change in research trajectories has occurred lately by exploiting, among others, omics approaches, which have contributed to shedding some light on the mechanistic processes leading to the positive or adverse effects of REEs repeatedly reported in the literature [141]. In addition, the valuable information gained on animal/human systems regarding the interaction of REEs with biological macromolecules can be extended to the understanding of presumably analogous molecular determinants in plants [143].

Three levels of influence of REEs have been called into play to explain the beneficial effects of these elements: metabolic, structural, and cytogenetic [100]. The metabolic level concerns the interaction (or interference) of REEs with metabolic pathways and the functioning of cell organelles, particularly the processes of assimilation (photosynthesis), and ionic and redox homeostasis [144]. The structural effects concern the adjustment (or alterations) of plant morphology, from the cellular (cell wall, organelles) to the tissue and organ level, i.e., effects related to plant morphogenesis. The cytogenetic level refers to modulations of the cell cycle, affecting particularly the frequency of cell divisions. Together with REE-modulated hormonal signalling, the combined result of REE actions is a measurable positive effect on plant growth and development, but also on the ability of the plant to counteract environmental stress. However, the same levels of interaction of REEs with plant processes and structures similarly relate to their toxic actions (Figure 6).

### 5.2. Interference of REEs with Ca^2^⁺-Mediated Signalling

REEs significantly influence molecular mechanisms in plants by modulating Ca^2+^ signalling pathways, interacting with CaM and altering the related protein phosphorylation dynamics. Ca^2^⁺ is a central secondary messenger in signal transduction in plant cells, essential for nearly all physiological and biochemical processes. La^3^⁺ is sometimes considered a “super calcium” because of its strong functional resemblance to Ca^2+^ [99,145]. In general, REE^3+^ cations possess a higher charge-to-volume ratio than Ca^2+^, granting them a competitive affinity for calcium-binding sites [13,146]. Consequently, La^3^⁺ ions can substitute for Ca^2+^ in various cellular processes, occupying extracellular Ca^2+^ sites and reducing Ca^2+^ influx, thereby lowering intracellular Ca^2+^ availability for downstream signalling [147].

When La^3^⁺ levels rise excessively, they may disrupt cell membrane Ca^2+^ channels and calcium-mediated processes that are essential for cellular regulation [98]. At high concentrations, La^3^⁺ forms strong coordination bonds with CaM: it combines with two Ca^2+^-binding sites, leading to a looser and more disordered CaM structure that restricts its normal functions [103]. Normally, CaM mediates Ca^2+^ signals by undergoing conformational changes upon Ca^2+^ binding, enabling it to interact with downstream target proteins, such as kinases and phosphatases. However, the high charge and coordination properties of La^3^⁺ allow it to bind to EF-hand motifs in CaM, inducing structural deformation of this protein. This alters the CaM affinity for Ca^2+^, affecting its capacity to activate essential enzymes like CaM-dependent kinases and phosphatases, thereby impairing signalling cascades, including pathways critical for plant growth and metabolic regulation subject to environmental changes [60,103]. Accordingly, the decreased CaM-dependent kinase activity at high La^3^⁺ concentrations results in diminished stress resilience [34,148]. For example, La^3^⁺ can reduce the phosphorylation rates of proteins involved in oxidative stress mitigation, potentially downregulating antioxidant defenses and sensitizing plants to environmental stressors [149]. Transcription factors regulating stress response gene expression may also exhibit altered phosphorylation, affecting pathways crucial for plant resilience [60].

The interference of REEs with Ca^2+^-mediated signalling actually already stems from the interaction of REE^3+^ cations with Ca^2+^ channels, owing to their comparable ionic radii (Figure 3). Particularly, REE^3+^ act as Ca^2^⁺ channel blockers, with consequences on Ca^2+^ fluxes in and out of the cytoplasm [150]. The application of Gd^3^⁺ as a Ca^2^⁺ channel blocker in *Arabidopsis thaliana* led to the inhibition of KIN gene expression and, subsequent, less effective cold acclimation [151]. Additionally, Gd^3^⁺ significantly inhibits Ca^2+^-selective, stretch-activated (SA) channels involved in the mechano-transduction of physical stimuli, such as touch. SA channels operate as voltage-gated rectifiers, allowing Ca^2+^ influx following the trans-membrane chemical potential gradient [152]. Gd^3^⁺ blocks the channel only from the cis-side, where Ca^2^⁺ typically enters, thereby obstructing the Ca^2^⁺ signalling crucial for touch responses, as observed in tendrils. In maize roots exposed to 50 mM Gd^3^⁺ for 24 h, the inhibition of SA channels resulted in an 80% reduction in xylem exudation, confirming Gd^3^⁺ as an SA channel blocker [87,153]. Beyond such channels, REEs affect other Ca-dependent enzymes, such as phospholipase D (PLD), where Tb^3^⁺ displaces Ca^2+^, thereby reducing PLD function and horseradish peroxidase activity [154].

With respect to the interference of REEs with Ca^2+^ signalling, Eu is an interesting and neglected case. In an experiment with *Amaranthus caudatus*, Zeng et al. proposed that Eu^3+^ enters cells through Ca^2+^ channels and, like the other REEs, it replaces Ca^2+^ in CaM, enhancing downstream phytochrome signal transduction, ultimately stimulating the synthesis of the betacyanin, amaranthin [13,155]. However, while Eu^3+^ and Ca^2+^ have a similar ionic radium (1.07 vs. 1.12 Å), Eu displays alternative valence, and it is conceivable that in the cell environment it could be reduced to Eu^2+^, which is larger (1.25 Å) than Ca^2+^ (Figure 2).

### 5.3. Effects of REEs on General Plant Metabolism

REEs significantly influence the production of metabolites in plants by modulating primary and secondary metabolic pathways. Changes in metabolite concentrations depend on adjustments of the oxidative balance, enzymatic activity, and gene expression. While the precise molecular mechanisms are still under investigation, evidence suggests that small amounts of REEs can act as elicitors, triggering signalling cascades that activate genes involved in primary and secondary metabolism [60,87,100,156].

The interference of REEs in phosphorylation and dephosphorylation cycles amplify the responses to stress, leading to the activation of transcription factors that enhance the expression of genes associated with metabolite biosynthesis [60,87,100,156,157,158,159]. The REE-induced increased production of specialized metabolites, like phenolics and alkaloids, has been reported in plants as different as *Salvia miltiorrhiza* (dicot), *Crocus sativus* (monocot), and *Taxus yunnanensis* (gymnosperm) [160,161,162,163,164,165].

REEs can also trigger oxidative stress in plants, as evidenced by increased levels of ROS and lipid peroxidation byproducts, such as malondialdehyde, a marker of cell membrane damage [166]. In response to oxidative stress, plants activate a defense system that includes the production of osmoprotectants (e.g., soluble sugars, proline, betaine) and both enzymatic and non-enzymatic antioxidant components to counteract the harmful effects of ROS [167]. It is possible that the oxidative stress induced by REEs could result in the enhancement of the plant’s resistance to environmental stress frequently reported in crops treated with low doses of REEs. Ce^3+^, in particular, increases proline levels, an amino acid with significant hydration potential, enhancing the plant’s resistance to water stress and improving the water use efficiency in *Salvia mirzayanii* [168]. In turn, proline mitigates oxidative stress by scavenging ROS and preventing lipid peroxidation [169]. Similarly, after applying Gd^3+^ treatment to *Medicago sativa*, the levels and metabolic activity of the plant’s osmoprotectants and antioxidants were enhanced. In line with the rise in the proline and anthocyanin content, the activity of their associated metabolic enzymes (e.g., ornithine aminotransferase [OAT], Δ^1^-pyrroline-5-carboxylate synthetase [P5CS], phenylalanine ammonia-lyase [PAL], and chalcone synthase [CS]) also increased [166]. A higher PAL activity also explains the increased phenolic compounds and, particularly, the flavonoid content in *Glycyrrhiza uralensis* and *Helianthus annuus* after exposure to REEs [161,170,171]. Another study demonstrated that the foliar application of low doses of La^3^⁺ can effectively reduce arsenic (As)-induced phytotoxicity in *Solanum nigrum* [172]. Increased tolerance is facilitated by activating several metabolic pathways, including amino acid, glucose, and flavonoid metabolism in plant roots. Furthermore, ascorbate and aldarate metabolism, caffeine metabolism, and phosphatidylinositol signalling pathways were significantly enhanced by La^3^⁺ treatment [172]. Furthermore, La^3^⁺ has been reported to increase the content of camphor, linalool, linalyl acetate, and lavandulol acetate in *Lavandula spica* flowers and leaves under oxidative stress conditions [173]. In *Triticum aestivum*, La^3^⁺ and Ce^3^⁺ exposure affects sucrose, fructose, and maltose levels, suggesting potential impacts on starch content, a crucial factor in regard to wheat grain yield and quality [140]. In *Catharanthus roseus* cell cultures, 50 mg of L^−1^ CeCl_3_ increases raubasine and polysaccharide production [174], while 5.8 mM of La(NO_3_)_3_ in *Taxus yunnanensis* cultures promotes taxol synthesis and release [175]. Other antioxidants enhanced by REEs include carotenoids. Ce^3^⁺ and La^3^⁺, for instance, have been observed to stimulate cell growth and carotenoid (crocin) production in Crocus sativus callus cultures [176].

In addition to non-enzymatic antioxidant molecules, antioxidant enzymes are also activated in response to REEs [13,60,87,100,159]; for example, in *Lemna minor*, Tb^3+^ (5–100 mg L^−1^) activates enzymes, such as superoxide dismutase, catalase (CAT), peroxidase (POD), and glutathione S-transferase, along with glutathione reductase and ascorbate peroxidase, which are essential components of the ascorbate–glutathione cycle [177]. These enzymes play a key role in detoxifying ROS and maintaining the cell redox balance [157,178,179]. However, the influence of REEs on the redox balance varies based on the concentration of the specific REE and the plant species. Low REE concentrations (<200 μM) typically activate antioxidant enzymes like CAT, POD, and PAL, whereas higher concentrations (>300 μM) can inhibit enzymatic activity, leading to high levels of oxidative stress [180].

Together with ROS-related metabolic effects, REEs also influence the synthesis and action of key phytoregulators, including jasmonic acid, abscisic acid (ABA), indole-3-acetic acid (IAA), and salicylic acid (SA), which regulate plant metabolism and development, as well as the plant’s adaptation to abiotic stress, allowing plants to respond to environmental stimuli locally and systemically [157,181]. However, it is very difficult to draw a general framework in regard to the effect of REEs on the synthesis of phytoregulators. Supplementation with La^3^⁺ in *Oryza sativa* and *Zea mays* has also been linked to increased levels of not only IAA and SA, but also gibberellins (GA) and cytokinins (CK) [157]. In *Zea mays*, 20 mg of L^−1^ La^3^⁺ enhanced GA and IAA levels, particularly when combined with UV-B exposure, suggesting that La^3^⁺ improves the plant’s tolerance to UV-B stress [182]. Foliar treatment with Tb^3^⁺ at levels ranging from 20 to 200 μM decreased the auxin and GA content, while increasing the ABA content in *Armoracia rusticana* [183]. In *Dendrobium densiflorum,* Nd^3+^ significantly increased the auxin levels as just mentioned in maize, but with minimal impact on the overall endogenous CK levels [184]. Conversely, the negative impact on the growth of *Limonium crithmoides* was associated with the disruption of PIN-dependent auxin transportation, which altered the auxin distribution and hindered primary root development [185]. Investigations using the transgenic marker lines, AUX1-YFP and PIN1/2/4/7-GFP, demonstrated a reduction in the auxin transporter levels in primary root tips following La^3^⁺ exposure, coupled with increased stabilization of the Aux/IAA protein, AXR3. These observations suggest that at high concentrations, La^3^⁺ interferes with PIN-mediated auxin transportation, limiting the availability of auxin transporters [185]. In general, the REE concentration plays a major role in determining the changes in phytohormone patterns, resulting in the contrasting results that can be associated with the hormetic action of REEs on plant metabolism.

### 5.4. Effects of REEs on Photosynthesis

The impacts of REEs on plant photosynthesis occur at multiple levels: chlorophyll stability, photochemical quantum yields, electron transport, and carbon assimilation [87,100].

A primary effect of REEs on chlorophyll is the substitution of Mg^2^⁺, which is crucial for light absorption and energy conversion. When REEs replace Mg^2^⁺, they alter the chlorophyll structure and stability, with downstream effects on photosynthesis depending on the REE type, concentration, and environmental conditions [186,187]. This substitution stems from some similarities between REE ions and Mg^2^⁺ in terms of coordination chemistry. Notably, REEs, like La^3^⁺ and Ce^3^⁺, with a higher positive charge than Mg^2^⁺, have a strong affinity for coordination sites within the chlorophyll porphyrin ring and can bind even more strongly than Mg^2^⁺ to pyrrolic nitrogen, leading to a slight distortion of the ring [132,188]. Moreover, lanthanide-substituted chlorophylls were reported to facilitate the formation of “double-decker chlorophylls,” in which the REE^3^⁺ cation is coordinated by two parallel porphyrin rings [189]. This double-decker structure exhibits greater rigidity compared to natural chlorophyll containing Mg^2^⁺, contributing to an enhanced molecular symmetry caused by the longer La–N bond length compared to Mg–N. The extended bond length alters both the geometry and overall stability of the molecule [186]. In contrast, chlorophyll *a* complexes with Y^3+^ adopt a single-layer structure, akin to standard chlorophyll *a*. This structural distinction is likely due to relatively short ionic radius of Y^3+^, a consequence of the lanthanide series contraction (Figure 3) [187]. Song et al. demonstrated experimentally that the Gibbs free energy changes associated with these substitution reactions indicated that lanthanides mono-chlorophyll *a* compounds are significantly more thermodynamically favorable than their bis-chlorophyll *a* counterparts. Furthermore, simulated electronic absorption spectra of lanthanides mono-chlorophyll *a* compounds revealed that the replacement of Mg^2+^ with La^3+^, Ce^3+^, and Tb^3+^ reduced the overall absorption intensity of chlorophyll [132].

REEs impact chlorophyll levels by inhibiting their synthesis and accelerating their degradation. Specifically, REEs interfere with the chlorophyll biosynthetic pathway by inhibiting key enzymes involved in the production of chlorophyll *a* and *b*. La^3^⁺ was shown to depress, by a ca. of 80%, the activity of δ-aminolevulinic acid dehydratase (δ-ALAD), a Zn-dependent enzyme essential for chlorophyll synthesis [190]. The inhibition mechanism, commented on by the authors in terms of the general cell redox imbalance, deserves further investigation, in particular, to verify whether La^3^⁺ directly competes with Zn^2^⁺. Furthermore, REEs accelerate chlorophyll degradation by inducing oxidative stress within chloroplasts, which elevates ROS levels and alters the photosynthetic machinery by degrading photosynthetic proteins. ROS are known to inhibit the synthesis of the D1 protein in the photosystem II (PSII) core and disrupt the repair cycle of PSII, thereby aggravating photoinhibition and photodamage [191,192]. For example, in *Oryza sativa*, exposure to 1.2 mM of La^3^⁺ reduced the amount of chlorophyll *a* by 35% and chlorophyll *b* by 30%, illustrating the negative impact of REE accumulation on pigment stability [193]. In *Spinacia oleracea*, exposure to 1.0 mM of La^3^⁺ caused a 25% reduction in the total chlorophyll, with chlorophyll b declining more than chlorophyll *a*, which adversely affected the chlorophyll complex stability and functionality [194]. Similar effects on the chlorophyll content of plants were recently reported by Jiang et al. in rice seedlings treated with mM concentrations of La^3^⁺ [188]. Moreover, the same authors, combining chlorophyll fluorometric and electrophoresis of thylakoid complexes, characterized the disruption of proteins integral to the photosynthetic electron transport: La^3^⁺ decreased the functionality of both the donor (Oxygen Evolving Centre, OEC) and acceptor sides of PSII (Q_B_ plastoquinone exchange site) (for a recent review on the PSII structure see [195]). Actually, all major thylakoid complexes (PSI, ATP synthase, Cytochrome *b*_6_*f*) decreased in terms of the relative amount and the supercomplexes formed by PSII and its light-harvesting complex (LHCII) were completely disintegrated [188]. Such interference slows the generation of ATP and NADPH, thereby limiting carbon fixation and, ultimately, reducing the net photosynthetic rate. Jiang et al.’s paper provides a good recent example of the potential of chlorophyll *a* fluorometry to disentangle the effects of REEs on the functioning of photosynthetic machinery. The responses reported by Jiang et al. repeat the detrimental, well-documented effects caused in general by heavy metal stress, which is a classical topic investigated using chlorophyll a fluorescence [188,196,197,198,199]. In fact, the effects determined by mM concentrations of REEs, though less documented, correspond to the non-specific damage caused to chloroplasts by many other heavy metals [198]. Chlorophyll fluorescence, both in the continuous excitation and amplitude modulated (PAM) mode, can contribute to damage characterization (for a review on fluorescence methods see [200,201,202,203]). For example, conclusions on La^3^⁺ effects on the PSII structure and function drawn by Jiang et al. were based on the increase in minimum fluorescence (*F*_0_) and the decrease in maximum fluorescence (*F_M_*), to infer the dissociation of LHCII from PSII and the blocking of electron transfer (see e.g., [204]). Additionally, the toxic effects of REEs on photosynthesis can be evidenced by the decline in relatively simple photochemical parameters, such as the *F_V_/F_M_* fluorescence ratio, which is a common proxy of the PSII maximum quantum yield, or the actual PSII photochemical efficiency (ΦPSII) under illumination [60,188]. More detailed information can be obtained from step-by-step analysis of the fast fluorescence increase upon exposure to a saturating light pulse, the so-called OJIP transient [199,205,206,207]. The first 2 ms long fast rise, called the O-J phase, is photochemical in nature and related to the effectiveness of light harvesting and exciton trapping in the PSII reaction centre, the LHCII-mediated connectivity of PSII units, the integrity of the PSII donor side, and the functionality of the PSII acceptor side, which is also related to the pool size of plastoquinones [205]. Accordingly, a relative increase in fluorescence at ca. 0.3 ms (K band) and 2 ms (J step) upon La^3^⁺ treatment was assigned to damage to the donor and acceptor sides of PSII, respectively [188]. The subsequent slower thermal phase or J-I-P relates to the efficiency of electron transport up to the final PSI acceptors [205]. The suppression of the I-P phase amplitude (30 ms) by a treatment with a high La^3^⁺ concentration was interpreted to be the result of an inhibited electron flow to the end acceptors at the PSI [188]. Complementary to the analysis of fast transients, fluorescence quenching analysis by PAM fluorimetry is another powerful tool to characterize REEs effects on photosynthesis. In luorescence quenching analysis, the focus is on slow changes (seconds to tens of minutes) in the fluorescence signals, which enable the calculation of parameters related to electron transport and light energy dissipation as heat; parameters such as non-photochemical quenching (NPQ) and quantum yields are very popular in stress response research in plants [203,208]. Albeit the interpretation of fluorescence signals is a matter of intense debate by chlorophyll fluorescence specialists [209,210,211,212,213,214,215], fluorescence remains a powerful tool to characterize, among others, the response of photosynthesis to REE exposure. Beyond the somewhat confirmatory knowledge on the toxic effects of REEs, which, on the whole, are very similar to those of other heavy metals, chlorophyll a fluorescence can make a major contribution to our understanding of the mechanisms that instead enable their beneficial effects at the level of sub-millimolar doses.

The enhancement of photosynthesis is part of the beneficial effect of REEs in plants. Numerous studies have found that the application of low concentrations of REEs significantly enhances the contents of chlorophyll *a*, chlorophyll *b*, and carotenoids [19,144,216]. Maintaining an adequate chlorophyll content, especially under stress, directly reflects the photosynthetic intensity of plants. In line with this, low REE concentrations increase the photosynthetic electron flow, reducing light energy dissipation in non-photosynthetic processes [216]. Ma et al. found that, in *Pseudostellaria heterophylla* under high light and temperature stress, the application of REEs to the leaves increased the efficiency of light energy absorption, conversion, and electron transfer, and alleviated the PSII photoinhibition caused by excess excitation energy. Moreover, they highlighted a better effect of La^3^⁺ than Ce^3^⁺. Particularly, based on OJIP transient analysis, the authors report enhanced activity at both the acceptor and donor side of the PSII, the latter gaining stability presumably because of the increase in the antioxidant enzyme activity [217]. However, further research is necessary to better understand the role of REEs on modulating the stability of the PSII. In a single study on the hyperaccumulator fern, *Pronephrium simplex*, Lai et al. reported that in chloroplast subfractions more than 50% of the REEs were located in the thylakoids and half of them were associated with the PSII [75]. Although this aspect was not investigated further, preferential binding of REE^3^⁺ to the PSII is conceivable because of the high density of negative charges at the stromal thylakoid surface due to the high level of phosphorylation of PSII core proteins and LHCII subunits [218,219]. In particular, the phosphorylated stromal loops of LHCII proteins are well-known to form bridges with Mg^2^⁺, leading to the “velcro-like” effect that stabilizes thylakoid appression in chloroplast grana [220,221,222]. In regard to beneficial ranges of REE concentrations, a limited substitution of Mg^2^⁺ in the phosphate-rich grana partitions with REE^3^⁺ could influence the many properties related to the degree of thylakoid appression: the rate of PSII repair [223]; the lateral segregation of PSII from PSI [224] and, conversely, the extent of the energy spillover as mediated by the PSII–PSI physical interaction at the grana margins [225,226]; the connectivity between PSII units on the thylakoid plane [227] and between vertically facing PSII supercomplexes [228]; and the long range diffusion of mobile electron carriers [229,230,231]. At present the influence of small amounts of REEs on thylakoid dynamics remains a completely unexplored field. Likewise, to date, no information is available about the interaction of REEs with PSI, but Lai et al., interestingly, reported that in *P. simplex,* only an almost negligible fraction of chloroplast REEs were associated with PSI. It can be speculated that PSI, which is dominated by Fe–S clusters [232] and is much less phosphorylated than PSII–LHCII [233], does not offer as many chances as PSII for molecular interactions with REEs. In the future, the elucidation of REE effects on PSI, either positive or negative, will benefit from P700 absorption kinetics analysis and electron paramagnetic resonance analysis of Fe–S clusters.

In addition to influencing electron transport, REEs directly impact RuBisCO, the enzyme responsible for CO_2_ fixation in the Calvin–Benson–Bassham cycle. RuBisCO requires Mg^2^⁺ for activation, and REEs can compete for Mg^2^⁺-binding sites, altering RuBisCO conformation, with effects that vary according to the concentration and environmental context [100,141,190]. At low concentrations, La^3^⁺ can promote RuBisCO activity and increase plant biomass; in vitro, the lanthanides ranked in regard to effectiveness as Ce^3^⁺ > La^3^⁺ > Gd^3^⁺ for enhancing RuBisCO activity [234]. Ce^3^⁺ increased RuBisCO activity by promoting the formation of a supercomplex between RuBisCO and RuBisCO activase: REEs could act as a “molecular bridge” between the two enzymes, potentially facilitating RuBisCO structural stability. RuBisCO would, therefore, be locked into an active “open” conformation [235,236]. However, excessive REE concentrations generally have negative consequences, also on RuBisCO activity [122,237].

REEs influence carbon assimilation, also affecting gas exchange and water-use efficiency in plants. At low concentrations, REE mixtures increase stomatal conductance (gs), which enhances CO_2_ uptake, carbon assimilation, and the overall photosynthetic performance of plants [142]. For example, preparations containing La^3^⁺ and Ce^3^⁺ increased the g_s_ and transpiration rates [238]. Ce^3^⁺ application improved the water-use efficiency in beans [239]. Sc^3^⁺ application had protective effects in rice under NaCl-induced salinity and PEG-simulated drought stress, enhancing the stomatal conductance, net photosynthetic rate, transpiration rate, and leaf water potential [169]. While the effects of REEs on stomatal dynamics could just be indirect, a recent study by Jiao et al. in *Arabidopsis thaliana* plants sprayed with La^3^⁺ solutions (15–80 mM) pointed to its interference with endogenous stomatal rhythms and gene expression. Increased stomatal opening and the higher expression of genes, such as OST1, OST2, PHYB, FT, CO, and CRY2, resulted in higher evapotranspiration rates. The authors proposed that upregulated genes may alter the activity of plasma membrane H⁺-ATPase in guard cells, facilitating turgor and stomatal opening, consistent with previous findings [240,241,242].

### 5.5. Effects of REEs on Cell Structures

REEs influence the stability of cell walls, membranes, and organelles critical for energy production and nutrient assimilation. In cell walls, ions like La^3^⁺ and Tb^3^⁺ were shown to replace Ca^2^⁺ in pectins, modifying the polysaccharide network’s rigidity and ion-exchange properties, which may weaken the plant’s defense against pathogens and abiotic stresses [19,100,243]. However, only very high doses of REEs can cause structural deformations. In *Glycine max*, no deformation occurred with La^3^⁺ at concentrations ≤80 mM, whereas in regard to exposure at 160 mM, it resulted in distinct depositions of the metal in the root and leaf cell walls, causing structural cell deformations. La^3^⁺ was uniformly distributed in root tissues, but concentrated in the xylem in leaves, suggesting tissue-specific and dose-sensitive responses [89]. As discussed in Section 4.3, the interactions of REEs with glycoproteins located at the interface between the cell wall and the plasma membrane, such as FLA17, influence endocytosis; however, additionally, they might also alter the cell wall’s stability [244].

The overall structural stability of the cell requires that the cell membrane is preserved. At high doses, REEs are responsible for detrimental effects on the cell membrane, primarily because of the excessive production of ROS, which oxidize proteins and lipids, modifying the membrane fluidity and permeability [245]. Among REEs, Ce^4+^ is a potent oxidant and exhibits higher toxicity than Ce^3+^ by inducing lipid peroxidation, disrupting the cell structure, and decreasing membrane fluidity [246]. ROS-mediated oxidation of aquaporins (plasma membrane intrinsic proteins, PIPs) further reduces membrane permeability and impairs water transportation, resulting in the loss of cell turgor, especially under stress [87]. REEs can also alter aquaporin activity by competing with Ca^2^⁺, essential for aquaporin activation [245]. In plants, aquaporin gene expression is tightly regulated at transcriptional and post-translational levels to maintain the water balance [247]. For example, in *Zea mays*, La_2_O_3_ nanoparticles (NPs) downregulate PIP expression, modifying the osmotic potential and restricting NP diffusion, leading to a reduction in the water-use efficiency [248]. In another report, in *Zea mays*, the La_2_O_3_ NP-induced suppression of PIP expression caused a reduction in water uptake, a decline in gs by up to 83%, and in transpiration by up to 86%; conversely, lignin biosynthesis genes were upregulated (ZmPAL, ZmC) [249].

Toxic concentrations of REEs alter mitochondrial and chloroplast function, deforming both organelles, reducing ATP production, and impairing energy availability for plant growth and resilience. In mitochondria, in addition to REE replacement of Ca^2^⁺, the REE-dependent increase in ROS levels further destabilizes mitochondrial membranes and increases their permeability transition pore (MPTP) activity, exacerbating cellular dysfunction [60]. Er^3^⁺ caused mitochondrial swelling, reduced membrane fluidity, and induced permeability changes, altering the MPTP and leading to a loss of mitochondrial function [250]. In the water plant, *Hydrocharis dubia*, treatment with 80 μM of La^3^⁺ caused notable structural alterations to chloroplasts (swelling, thylakoid disorganization), mitochondria (decreased cristae density, swelling), and nuclei (disrupted envelope, disorganized nucleolus) [251]. At higher concentrations (160 μM La^3^⁺), the damage intensified, with further chloroplast swelling and disintegration, as well as chromatin clumping and nuclear vacuolation, indicating a dose-dependent impact on the ultrastructure [251]. Similarly, in *Glycine max*, La^3^⁺ exposure disrupted the chloroplast and mitochondrial ultrastructure, impairing the photosynthetic efficiency and cellular respiration of the plant [89,193]. Hu et al. and Jiang et al. also reported the destruction of the chloroplast ultrastructure in the context of more general cell alterations in *Oryza sativa*, but the quality of the transmission electron micrographs in both reports was too low to draw any reliable conclusions about the REE effect on the chloroplast structure. However, we can extend to REEs a concept proposed by Solymosi and Bertrand that, apart from a few specific symptoms, all heavy metals tend to disorganize the chloroplast structure in a similar way [252]. Therefore, they conclude that chloroplast alterations are caused by unbalanced metal distribution within the organelle rather than a metal-specific effect. The same concept may also be valid for mitochondria. More interesting but also definitely more challenging, would be to test whether there are specific ultrastructural effects of REEs in regard to the beneficial dose levels. The problem should be approached by applying ultrastructural morphometrics, also assisted by advanced electron microscopies [253], deep learning-based methods for plant organelle phenotyping [254], and newly introduced parameters to identify specific properties, such as “grana irregularity” in chloroplasts [255,256].

### 5.6. Effects of REEs on Plant Growth, Development, and Cytogenetics

The influence of REEs on seed and plant development varies significantly depending on the concentration, plant developmental stage, and species. Generally, low concentrations of REEs positively affect germination, early growth, root development, vegetative structure, and flowering and fruit and seed production, whereas higher concentrations hamper the same processes [100,135,142,257].

The effects of REEs on seed germination often involve interactions with the phytohormones, such as ABA and GA, which regulate germination initiation and progression [34,258]. Appropriate REE concentrations have been shown to enhance germination and seedling growth [134,259,260]. For example, in *Oryza sativa*, Ramírez-Olvera et al. observed that seed germination increased from 6.4 to 36.2% in response to 12 μM concentrations of Ce^3^⁺ compared to untreated controls. However, REEs often impact germination speed more than the overall germination percentage [105,133,259,260], and this seems related to their role in activating enzymes like proteases, which are necessary for mobilizing seed nutrient reserves [107]. However, germination is not always promoted by REEs; in fact, the impact of REEs on seed germination also depends on environmental factors. Generally, the responses are linked to the chemical speciation of REEs, as hydroxide forms prevalent at an alkaline pH are less bioavailable and less toxic [19]. Species-specific differences were also observed: rice and soybean seeds germinated better in the presence of La^3+^, while LREEs inhibited wheat and corn germination [90,261,262,263].

After germination, REEs impact root and shoot growth, as well as flowering, when supplied at concentrations as different as 0.4–150 mg kg^−1^ [140,259,264,265]. Drobkov first reported enhanced pea yields with La^3+^, and subsequent studies in Chinese crops have confirmed the hormetic effects of REEs on plant growth and productivity [134,266,267]. These effects were observed with single or mixed REEs, promoting growth in various species, including the REE-accumulating fern, *Dryopteris erythrosora* [268].

In roots, low concentrations of REEs stimulate cell elongation and root hair proliferation, enhancing water and nutrient uptake. Increased root length has been observed in *Arabidopsis thaliana* (39.2% increase) treated with 10 μM of Ce(NO_3_)_3_ [269]. This effect is mediated by enzymes like nitrate reductase, which promotes nitrogen assimilation, critical for protein synthesis [270]. REEs also enhance the structural integrity of roots by increasing lignin deposition in cell walls, improving their resistance to mechanical and environmental stresses [19]. For instance, La_2_O_3_ nanoparticles increased the lignin content by 1.5-fold in roots, leading to early apoplastic barrier formation [249]. *Arabidopsis thaliana* exposed to 0.5 μM of Ce(NO_3_)_3_ and La(NO_3_)_3_ showed not only increased root growth, but also shoot development and flowering induction [269]. In *Zea mays*, treatments with Ce(NO_3_)_3_·6H_2_O (57.39 mg L^−1^) and La(NO_3_)_3_ (34 mg L^−1^) enhanced shoot dry matter and overall growth [87]. Eu, in the form of Eu(NO_3_)_3_, improved germination, as well as root and leaf growth, in *Secale cereale*, influencing nutrient uptake and distribution [271]. In *Lactuca sativa*, the application of Nd^3+^ at a concentration of 2.885 mg of Nd·L^−1^ enhanced shoot height, leaf area, and plant biomass, supported by increased foliar concentrations of N, P, and K [272].

The growth-promoting effects of REEs may result from improved nutrient uptake, enhanced photosynthetic pigment synthesis, and mobilization of energy reserves by phytohormones (GA, IAA), collectively boosting plant metabolism and growth. As reported in Section 4.3, the interference of REEs with several plasma membrane transport mechanisms can result in enhanced mineral uptake. Nevertheless, the precise mechanisms behind specificities related to the individual accumulated minerals that are taken up by the plant remain unclear [13,19,60,100,135]. For example, Xie et al. demonstrated that La^3+^ has variable effects on element content in different parts of *Oryza sativa*. At low concentrations (0.05–0.75 mg L^−1^), La^3+^ increased the uptake of Cu, Fe, and Mg in the roots, as well as Cu, Ca, P, Mn, and Mg in grains. However, at higher concentrations (9–30 mg L^−1^), La^3+^ reduced the uptake of Ca, Zn, P, Mn, Fe, and Mg in grains, as well as Ca, Mn, and Mg in straw [273].

At toxic concentrations, the general principle for causing altered elemental compositions with reduced amounts of essential minerals is competition between REEs and other elements for the same transport mechanisms. The most characterized competition between REEs is with Ca^2+^: on one hand, the competition directly reduces the Ca^2^⁺ uptake, on the other hand, REE^3+^ acts as a channel blocker, further exacerbating the deficiency [100]. Accordingly, Kobayashi et al. highlighted that La^3^⁺ interferes with Ca^2^⁺ channels and inhibits root growth in *Arabidopsis thaliana,* acting with a mechanism similar to verapamil, a phenylalkylamine [274]. La^3^⁺ and Gd^3^⁺ exposure can also lead to P deficiency by precipitating phosphates. As a consequence, available P for the plant decreases, primary root growth is inhibited, while in the response to the P deficiency, root hair and lateral root development is induced, along with the upregulated expression of the phosphate transporters, AtPT1 and AtPT2 [275,276]. In addition to Ca^2^⁺, competition for uptake also occurs with other metal ions, for e.g., it was suggested that Gd^3^⁺ also interferes with the uptake and transport of Fe and Mg, leading to a deficiency impacting on chlorophyll synthesis and, consequently, leaf chlorosis [277]. In general, the deficiency symptoms seem to follow a scale of increasing toxicity with the molecular mass and, therefore, they are more severe with HREEs than LREEs [277]. An additional mechanism to explain REE-induced nutrient deficiency refers to the negatively charged molecules exposed on the plasma membrane surface, which can bind REEs; as a side effect, an abnormal distribution of other elements can occur on the membrane surface [278]. This irregular interaction can also disrupt the membrane potential gradient, impeding the mineral uptake that depends on active transport mechanisms [13].

Examples of REE benefits on plant growth under a range of environmental stresses have been reported. La^3+^ increased the biomass in *Solanum nigrum* under Cd and Pb contamination by 24.68% [279]. Similarly, Ce^3+^ reduced Cd toxicity in rice seedlings, increasing the chlorophyll content and preserving the chloroplast structure [280]. La reduced the viral symptoms in *Nicotiana tabacum* seedlings exposed to the cucumber mosaic virus [281]. Sc^3+^ alleviated drought-induced damage in rice by enhancing glutathione activity and stabilizing the gas exchange [169]. REEs also improve water-use efficiency, with La^3+^ enhancing stomatal resistance and osmotic adjustment in *Hordeum vulgare*, leading to a 21% improvement under drought conditions [84]. Furthermore, REEs help plants respond to cold stress, by reducing cell membrane permeability, maintaining cell stability at low temperatures [263].

High concentrations of REEs negatively impact germination and plant development, with their toxicity depending on the REE dose and plant species [282]. For instance, La^3+^ at 30 mg L^−1^ increased the germination of *Salvia miltiorrhiza* seeds, but at 100 mg L^−1^, had the opposite effect, illustrating its hormetic action [259]. Delayed or aberrant development of roots and shoots has been reported in a wide range of crops exposed to different REEs at concentrations of 0.2–2 mM, such as in *Triticum aestivum* [141,283], *Oryza sativa* [284], *Brassica juncea* [190], and *Camellia sinensis* [285]. In *Lemna gibba* exposed to 11.5–20 mg of L^−1^, REE decreased growth was accompanied by increased mortality [286]. In *Glycine max* treated with La^3^⁺ and Ce^3^⁺ at concentrations of 200 and 2000 mg L^−1^, alterations were observed in the epidermal wax [287]. The cells surrounding the affected areas collapsed, and the extent of the leaf damage was directly linked to the accumulation of REEs on the leaf surface [13,288].

In general, the altered plant morphogenesis is due to the already analyzed interference of REEs with physiological and biochemical processes, causing ion imbalances, oxidative stress, and several cellular dysfunctions. However, the interference of REEs with cytogenetic processes has also been documented and is relevant to understand the morphogenetic alterations [248,289]. As for other processes, REEs have a hormetic effect on mitosis. At low doses, REEs can increase the mitotic index, contributing to biomass accumulation [290]. Conversely, the opposite occurs when REE concentrations are too high. In *Allium cepa*, high La^3^⁺ and Ce^3^⁺ levels reduced the mitotic index and increased the presence of aberrant cells, disrupting cell division [146]. Nuclear abnormalities were also reported in soybean exposed to high La^3^⁺ levels [89]. REE-induced toxicity has indeed been associated with DNA damage and induction of chromosomal abnormalities: sticky chromosomes, wandering chromosomes, and chromosomal bridges were observed in *Allium cepa* root cells [291]. These findings suggest that high levels of REEs may disrupt mitotic spindle microtubules, leading to impaired chromosome segregation. Similar to other heavy metals, such as Cd [260,292], chromosomal aberrations and reduced mitotic indices contribute to the inhibition of root growth. Moreover, negative effects on other cell-division cell substructures, particularly the phragmoplast, could explain the occurrence of binucleate cells, as observed in soybean treated with La^3^⁺ [89].

## 6. Conclusions and Future Directions for Research

Pivotal in regard to numerous technological and agricultural advancements, REEs are increasingly becoming a focus of environmental and biological scrutiny owing to their dual nature as beneficial, yet potentially hazardous, entities within ecosystems. Their expanding prevalence, driven by anthropogenic activities, necessitates a profound understanding of their dynamics in soil–plant systems and the broader environment.

The mobility and bioavailability of REEs in terrestrial matrices are governed by intricate interactions involving soil chemistry, organic amendments, and competitive interactions with other elements. Speciation, a process influenced primarily by the soil pH, cation exchange capacity, and organic matter content, determines the extent of REE uptake by plant roots and their subsequent translocation within plant tissues. The rhizosphere, enriched with root exudates and featured in microbial interactions, significantly modulates REE mobility. Fractionation processes further delineate accumulation preferences, with LREEs exhibiting greater affinity for specific soil and plant compartments compared to their heavier counterparts.

Physiologically, REEs demonstrate a biphasic impact on plants characterized by hormesis. At low concentrations, REEs enhance physiological processes, such as photosynthesis, nutrient assimilation, and stress resilience, while at higher concentrations they induce toxicity, oxidative stress, and disruptions in calcium-mediated signalling pathways. These effects highlight the necessity for careful threshold management to mitigate their deleterious impacts on enzymatic activity, cellular integrity, and overall plant health. However, many aspects of the REE–plant interaction are still partly or completely unclear and warrant further research. Among others, we propose that the following topics deserve special attention:

(a) The *simultaneous chemical interaction of REEs* with soil phosphates, sulfates, and carboxyl groups of pectins at the root surface, and whether the plant cell is able to actively modulate it, for example, through pectin (de)methylation;
(b) *REEs and mineral nutrition*, whether a general framework can be developed to understand how REEs can improve the uptake of specific microelements, including a probable cooperative role with mycorrhizal fungi;
(c) Whether and how *REE fractionation* inside the plant is dependent on REE redistribution through the phloem and is related to different mechanisms of phloem (un)loading;
(d) The *Eu anomalies* in plant organs relative to the soil, whether it is related to the redox chemistry of Eu within the plant cell;
(e) The *central role of ROS* generated by REEs inside the plant cell, from which most of the beneficial changes could ensue;
(f) The *REEs as photosynthetic membrane stabilizers*, disentangling whether it is an indirect (antioxidant induction) and/or direct (photosystem stability or other) effect.

Advancing molecular, biophysical, and omics technologies can provide critical insights into the mechanisms of REE interactions within biological systems, paving the way for targeted applications and risk mitigation. Advancement in the knowledge of REEs in the soil–plant system is of great importance, for example, in agricultural contexts, where REEs hold potential for enhancing crop yield and resilience, and, at the same time, their application is overshadowed by environmental concerns. In fact, the accumulation of REEs in soils, often a result of industrial and agricultural activities, poses risks of bioaccumulation and ecological toxicity, including disruptions to soil microbial communities and adverse effects on terrestrial biota. These challenges underscore the importance of integrative management strategies. From a sustainability perspective, mitigation approaches, such as phytoextraction, recycling, and the development of REE alternatives offer promising avenues.

In conclusion, while the usefulness of REEs in modern technology and agriculture is undisputed, their environmental and biological repercussions necessitate a balanced approach. Addressing these challenges demands interdisciplinary strategies that align technological progress with ecological stewardship, ensuring that the benefits of REE utilization are realized without compromising environmental integrity or human health.

## Data Availability

No new data were created or analyzed in this study.
